# Crossing the Death Valley to Transfer Environmental Decision Support Systems to the Water Market

**DOI:** 10.1002/gch2.201700009

**Published:** 2017-04-10

**Authors:** Manel Poch, Joaquim Comas, Ulises Cortés, Miquel Sànchez‐Marrè, Ignasi Rodríguez‐Roda

**Affiliations:** ^1^ Laboratory of Chemical and Environmental Engineering (LEQUIA) Universitat de Girona Campus de Montilivi s/n 17071 Girona Catalonia Spain; ^2^ Catalan Institute for Water Research (ICRA) Parc Científic i Tecnològic de la Universitat de Girona Emili Grahit 101‐17003 Girona Catalonia Spain; ^3^ Knowledge Engineering and Machine Learning Group (KEMLG) Computer Science Department Universitat Politècnica de Catalunya‐BarcelonaTech Jordi Girona 1‐3 08034 Barcelona Catalonia Spain; ^4^ Barcelona Supercomputing Center Jordi Girona s/n 08034 Barcelona Catalonia Spain

**Keywords:** adoption, death valley, development, EDSS, water

## Abstract

Environmental decision support systems (EDSSs) are attractive tools to cope with the complexity of environmental global challenges. Several thoughtful reviews have analyzed EDSSs to identify the key challenges and best practices for their development. One of the major criticisms is that a wide and generalized use of deployed EDSSs has not been observed. The paper briefly describes and compares four case studies of EDSSs applied to the water domain, where the key aspects involved in the initial conception and the use and transfer evolution that determine the final success or failure of these tools (i.e., market uptake) are identified. Those aspects that contribute to bridging the gap between the EDSS science and the EDSS market are highlighted in the manuscript. Experience suggests that the construction of a successful EDSS should focus significant efforts on crossing the death‐valley toward a general use implementation by society (the market) rather than on development.

## 1 Introduction

New environmental challenges are qualitatively different from those of the recent past. The increasing understanding of environmental problems, the availability of more accurate modeling techniques, the considerable amount of deployed sensors, and the overwhelming evidence of environmental strain and global economic issues caused by the extensive pressure on resources exerted by the demands of growing populations indicate that environmental systems composed of multiple interacting agents or variables that cause emergent behavior require complex management strategies. In addition, improving environment‐friendly practices related to the use and/or reuse of natural resources to minimize detrimental effects on the natural environment has become a prominent issue. Therefore, increasingly advanced and sophisticated management tools capable of integrating heuristic knowledge and quantitative and qualitative information are required.

The initial concept of a decision support system (DSS) emerged in the business, economics, and management sciences. A DSS is an intelligent information system that reduces the time in which decisions are made within a domain and improves the consistency and quality of these decisions.^1^ DSSs use a combination of models, analytical techniques, and information retrieval to help develop and evaluate appropriate alternatives;^2^, ^3^ and such systems focus on strategic decisions and not operational ones. More specifically, a DSS should contribute to the reduction of the uncertainty faced by managers when they need to take decisions regarding future options.^4^ Similar definitions can be found in ref. 5, 6

Environmental decision support systems (EDSSs) are a class of DSS that demonstrates all of the capabilities of a DSS on an environmental field scale.^5^, ^7^, ^8^ EDSSs have been increasingly applied in more intense and efficient ways, and an increased number of systems have been developed. A metric of their increasing implementation is that more than 3300 references have been cited on this topic in specialized journals over the last ten years (search on SCOPUS on February 2016).

The quality and quantity of available water resources is decreasing because of the intensive use of surface and groundwater by cities, industry and agriculture, and the associated impacts of the point pollution and diffuse discharge of the generated wastewater.^9^ The optimal and integrated management of water resources is a paradigmatic example of circumstances in which an EDSS can play an important role by integrating data from different origins and with different quality with infrastructures managed by several public and private actors under different legal frameworks with a final goal of preserving the local (and global) environment while fulfilling the users' requirements.

### 1.1 EDSS Development and EDSS Use and Transfer

There are two main aspects to consider once an EDSS has been selected to resolve an environmental problem: the development of the tool and the use and transfer of the tool to the market.

Regarding development, the problem must be clearly identified, all of the available data and knowledge must be gathered and filtered, the necessary tools to infer the solution must be integrated, and all alternatives must be compared and ranked in a multi‐criteria framework. In addition to considering the technical aspects, the environmental, economic, and social aspects must also be appropriately considered in relation to the restrictions caused by a limited (and often public) budget.

Several methodologies have been proposed in the literature for the development of EDSSs,^10^, ^11^, ^12^, ^13^, ^14^, ^15^, ^16^ and many EDSSs have been deployed. These methodologies propose the basic steps, tools and main concepts to be considered when developing an EDSS.

The major challenges that have been identified in the development step are as follows:

- Knowledge acquisition and maintenance
- System validation
- Model integration and interoperability
- Uncertainty analysis and management
- Spatial and temporal reasoning

Historically, because of the complexity of the development step, most efforts have been devoted to this technical phase. In our opinion, a significant degree of maturity has been reached in the development of EDSSs, which are considered well‐established and reliable tools. The results obtained for the development of the basic concepts and tools and their integration and application to specific problems have been satisfactory in terms of applicability and usability, although work is still required for the development of new tools and applications.

For the second step, the use and transfer of the EDSS, the procedures and the relevant factors are not clear enough to guarantee the success of the developed tool. This finding is common for a number of innovative products, which face considerable barriers when crossing the death valley from an academic conception to a viable product in the market. Such barriers are more formidable for EDSSs because of the particularities of the environmental problems, tools, and potential users.

In this step, the following must be identified: the source of the problem; the associated effects of the problem; the individuals tasked to manage the problem; when the problem must be managed; and the implications if the problem cannot be solved (e.g., the solution suggested by the EDSS was not successful or was not applied). Additionally, it is necessary to identify who the final user is, whether the problem must be resolved once or upon frequent reappearances, and whether the user must confront other environmental problems when considering aspects of reuse, including the reuse of knowledge and the inference tool.

Regarding the challenges identified by McIntosh et al.,^17^ we suggest that the following challenges are the most important:

- Identifying problem ownership and engaging the end‐user
- encouraging longevity and financial sustainability
- evaluating the system

The main goal of this paper is to identify the key aspects to improve the technology transfer of EDSSs to the water market. In Section 2, four case studies of an EDSS developed by the authors for the wastewater treatment field are described and analyzed. The principal analysis and discussion are provided in Section 3, and the conclusions are detailed in Section 4.

## 2 Analysis of the Developed EDSS

This section describes and compares four EDSS case studies developed by the authors and applied to the water treatment domain. These four cases represent different common situations in the EDSS development domain. Two of them correspond to EDSS for planning where response times are not critical while the other two supports the operation of water infrastructures where process dynamics is very important. Besides, one of them was commissioned by water authorities, two were initially conceived from the academic world, with the intention to be adopted later in the market, i.e., willing to cross the death valley, and the last one was envisaged totally in collaboration between academia and one company of the water sector.

For each EDSS, both the development and use and transfer steps are illustrated, aiming at highlighting the most relevant features (**Table** **1**). The first two cases (PSARU and ATL) are only briefly described as they were previously presented in Poch et al.^14^; however, two new complementary examples (NOVEDAR and COLMATAR) are described in more technical detail. Information related to the objectives and development (e.g., identifying the environmental problem, determining whether the problem is market or research driven, and assessing the funding scheme, knowledge engineering consortia characteristics and commercial perspectives), as well as the evolution in terms of use and transfer to market of the four EDSSs are highlighted in this section to provide a basis for a final comparison and discussion in Section 3.

**Table 1:** Summary of the main features of each of the developed EDSSs

	PSARU	ATL	NOVEDAR	COLMATAR
Domain	Wastewater	Wastewater	Wastewater	Wastewater
Level of decision	Planning	Operation	Planning/design	Operation/control
Commissioned by	Water authority	University	Consortium of 11 universities	University and company
Developed by	Consortium of universities	Two universities	11 Universities	University and company
Funded by	Water authority	Research projects	Research project	Research and transfer of technology projects
Time for development	Three years	Ten years	Four years	Six years
Methodology for development	Poch et al.^14^	Poch et al.^14^	Poch et al.^14^	Poch et al.^14^ simplified
Reasoning tools	Rule‐based system	Rule‐based system + case‐based reasoning + modeling	Knowledge‐based system	Rule‐based system and mathematical model
Knowledge update/maintenance (incl. funding)	No update	Commercial spin off	Private company	University and private company
Current status	Unused	Commercial	Crossing the death valley	License agreement
Stakeholder implication	Strong	Weak	Under requirement	Medium
Validation	Expert‐based and stakeholders	Pilot and full‐scale	Expert‐based and pilot and full scale	Pilot and full scale
Research to market strategy	Null	Spin off	Licensing protocol	Patent and license agreement

### 2.1 PSARU EDSS

### 2.1.1 Objectives and Development

The PSARU EDSS was commissioned by the Catalan Water Agency to a consortium of research groups with the objective of selecting the most appropriate wastewater treatment and disposal system for 3500 communities with less than 2000 inhabitants in Catalonia.

A consortium of four environmental engineering research groups from different universities and the Spanish Scientific Council led by the University of Girona was established to acquire and systematize the required knowledge and develop a system capable of reproducing the reasoning process of a group of experts facing the complex situation in question.

The methodology of Poch et al.^14^ was used to build the EDSS. This particular application posed challenges in the following three sustainability dimensions.

- Social: Small communities have a strong perception of their sanitation treatment processes.
- Economic: Applications that pertain to a small percentage of the population (5%) but have a relatively high cost because of the scaling factor (i.e., the cost per cubic meter treated is significantly higher than for a large population).
- Environmental: Many target communities located in protected and/or tourist areas.

The project development involved intensive field and desk work, which required continuous interactions with the end‐users (the Catalan Water Agency) and with the stakeholders and the local authorities.

The PSARU EDSS provided satisfactory results with regard to assessing and validating the most appropriate sanitation systems in all of the predicted communities in Catalonia, which was the principal outcome.

### 2.1.2 Use and Transfer Evolution

After the success of the first project, two new goals were established: use the PSARU EDSS in future implementation phases of the Catalan Sanitation Plan and use the base (KB) and the evaluation criteria contained in the EDSS to resolve similar issues in other European regions required to implement the European Directive 91/271. However, none of these goals were achieved. Regarding the first goal, an economic crisis delayed the implementation of the Catalan Sanitation Plan, and subsequent changes occurred in the administration management; therefore, the product was relegated to a personal choice, and continuity was not observed. Regarding the second goal, although multiple contacts were established and negotiations were performed, neither the research groups nor the administrations were able to transfer and apply the system to other regions facing similar issues. We believe that such circumstances represent a general issue for many EDSSs that are designed and used for a specific requirement, which justified its construction, and then no longer used after the initial implementation.

#### 2.2 ATL EDSS

#### 2.2.1 Objectives and Development

The ATL EDSS originated in the mid‐1990s when two environmental engineering and artificial intelligence research groups joined forces to optimize the operation of the biological processes of municipal wastewater treatment plants (WWTPs). Initially, the work was performed conceptually by developing specific decision trees in the context of conventional expert systems.

Once a set of decision trees was generated to diagnose and resolve operational problems in an activated sludge system, a second phase was begun to build a complete EDSS. Among the modules, a case‐based reasoning system^18^ and a mechanistic model were developed. The same methodology as that used by Poch et al.^14^ was followed, and the resulting tool was implemented in a full‐scale facility (Granollers, NE Spain) that treated 35 000 m^3^ d^−1^. The ATL EDSS supervised the process for ten months, diagnosed problems and identified the causes on the same day (70%) or several days in advance (30%) of the problem.^19^

#### 2.2.2 Use and Transfer Evolution

Based on the satisfactory scientific results observed for EDSSs, the next goal was to adapt the tool for implementation in other facilities with similar wastewater treatment technologies. At this point, a problem arose as to whether this goal should be part of a research project or performed in collaboration with public or private companies. The proposed solution was the creation in 2003 of a spin‐off company named SISLtech which was tasked with proceeding with the further development and commercialization of the ATL EDSS, with the researchers signing on as entrepreneurs. However, the protocol for the practical implementation of the ATL EDSS to any WWTP was complex and long, and it required too much effort from the developers (researchers) and the end‐users (facility managers). Therefore, the commercial initiative to develop the ATL EDSS was considered unsuccessful.

In 2009, a key shift in orientation occurred. The owner of the WWTP where the first version of ATL EDSS was validated identified a market opportunity for product commercialization and subsequently bought most of the shares of the spin‐off company. A new manager took control as director of the company, and the five initial entrepreneurs became the research unit behind the scenes and developed and refined aspects related to the knowledge engineering tasks, although they did not have an influence on the commercialization. The first and main task of the company was the adaptation of the product (and its implementation protocol) for actual market needs. The complexity of the tool was high as a result of the development methodology, which represented a considerable opportunity from an academic perspective for the exploration and publication of new approaches. From a commercial perspective, however, the system had to be drastically simplified for practical implementation. Thus, an optimal cost/benefit balance had to be found, which was an aspect that the researchers did not consider. Presently, the spin‐off SISLtech has successfully implemented an evolved version of the ATL EDSS to more than one hundred plants in ten countries treating more than a million of cubic meters a day.

### 2.3 NOVEDAR EDSS

#### 2.3.1 Objectives and Development

The NOVEDAR EDSS was developed in the framework of an academic research project with the goal of selecting the optimal integration of both conventional and innovative technologies for the treatment of specific wastewater in a specific location. The project started in 2007, and the consortium involved 11 universities, which had the general goal of defining the concept of a 21st century WWTP. The framework was organized in four main work units: (i) study and optimization of innovative technologies for wastewater treatment and recovery strategies; (ii) process modeling for performance and cost; (iii) development of economic, environmental, technical and social evaluation criteria; and (iv) integration of knowledge into an EDSS to select the optimal combination of conventional and innovative technologies for a specific scenario according to specific evaluation criteria.^20^

Five steps were followed to build the NOVEDAR EDSS according to the methodology of Poch et al.^14^:

##### 2.3.1.1 Problem Identification :

The selection of an optimal combination of technologies for a WWTP configuration is a complex problem. A single solution is not sufficient, and a combination of different technologies for the water (pretreatment, primary treatment, secondary treatment, and advanced treatment) and the sludge lines (thickening, enhanced digestion, and dewatering) is necessary. The NOVEDAR EDSS included 250 different existing technologies and had a large number of potential alternatives to compare. In addition, the criteria used to evaluate the suitability of the design became increasingly elaborated. The achievement of adequate effluent water quality levels was not the sole consideration, and additional aspects, such as the operational safety, cost (with special attention to energy requirements), and the environmental impact of the plant with respect to greenhouse gases and emissions, also had to be considered.

##### 2.3.1.2 Data and Knowledge Acquisition :

The data required to build the knowledge base of the EDSS was extracted from interviews with experts within the NOVEDAR Consolider research project from 11 universities, project engineers from 29 water companies, and experts from 14 public wastewater treatment authorities. A scientific and technical literature review was also used.

##### 2.3.1.3 Model Selection :

The proposed knowledge model was based on a hierarchical decision approach that reduced the complex design problem into a series of issues that were easier to analyse and evaluate. For each of these issues, three levels of abstraction were defined (units, sub‐metaunits, metaunits) to modify the degree of engineering detail and facilitate the decision making of each design step.^20^

##### 2.3.1.4 Model Implementation :

WWTP alternatives were generated by the interaction between two KB. The first KB included the main features of the different treatment technologies, whereas the second KB contained information on the degree of compatibility among the different technologies. Both KBs were linked to other databases with additional information on legislation and environment‐related issues. These characteristics were described using an integrated modeling approach through mechanistic process equations, cost models, life‐cycle assessments, and expert and bibliographic knowledge. The combination of the wastewater treatment technology properties, network structure model, decision trees, and recursive evaluation method with a multi‐criteria decision analysis allowed for (i) the synthesis of multiple flow process diagrams, including different treatment schemes; and (ii) the analysis of these diagrams from an environmental, economic, social, and technical perspective. Next, a multi‐criteria decision method selected the sequence of unit processes that maximized the degree of satisfaction of the different objectives by using multi‐criteria and sensitivity analysis tools.^21^, ^22^

##### 2.3.1.5 Model Validation :

The software was verified and validated by different research groups of the NOVEDAR Consolider research project and the Water 2020 Cost‐Action program (ES1202‐110614‐044095) and engineers from companies participating in the project. The results were considered satisfactory.

##### 2.3.2 Use and Transfer Evolution

Maintenance of the KB was the first and presumably most critical issue identified. New knowledge was generated from basic and demonstration research projects, particularly those related to innovative technologies for wastewater treatment and quantifying the evaluation criteria. Once the NOVEDAR Consolider research project was completed, additional funding was not available to update the NOVEDAR EDSS knowledge base; therefore, the tool could have quickly become obsolete. The consortium thought that the best method of ensuring the maintenance of the EDSS was to transfer the system to the market and receive funding from users. A different commercial strategy was used in this case. NOVEDAR EDSS was presented to several water companies, and one company was interested in reaching an agreement for license commercialization. The intellectual property rights (IPR) were shared and distributed between the universities that developed the system.

Negotiations with the company lasted for more than a year. A protocol for evaluating innovative projects by different departments of the company (processes, engineering, legal, computing, commercial, etc.) was strictly followed. Thus, the company had to verify interest in and the reliability of the NOVEDAR EDSS, but it also had to evaluate the cost of adapting the product to market needs. Similar to the ATL EDSS, the NOVEDAR EDSS was scientifically sound and complete but not ready for commercial use.

### 2.4 COLMATAR EDSS

#### 2.4.1 Objectives and Development

The main objective of the COLMATAR EDSS was to improve the integrated control of the biological and filtration processes of membrane bioreactors (MBR) for wastewater treatment in terms of the effluent quality and operational costs. The COLMATAR EDSS was initiated in 2006, and it was based on academic work and the funding of three research projects obtained in public competitive calls for research. From the onset, several wastewater practitioners identified the relevance of the proposal and showed a potential interest in collaborating. Specifically, a water engineering and construction company became fully committed to the development of the EDSS at an early stage.

Again, the protocol defined in Poch et al.^14^ was followed to build the COLMATAR EDSS.

##### 2.4.1.1 Problem Identification :

MBRs were an emerging technology for municipal and industrial wastewater treatment and the MBR global market was growing, especially in regions where water was scarce or water reuse required high‐quality reclaimed water. However, the high‐energy consumption rate of MBRs related to membrane cleaning via air scouring and limited knowledge or experience in MBR design and operation posed an obstacle to water authorities and companies that were considering committing to a definite implementation. In addition to the complex management of biological processes, the use of MBRs includes considerations for filtration processes and guarantees of adequate removal efficiencies with minimal fouling rates and operational costs.

##### 2.4.1.2 Data and Knowledge Acquisition :

The data and knowledge required to develop the COLMATAR EDSS were mainly acquired through the processing and interpretation of empirical data from experiments at different scales. The EDSS was tested on both laboratory^23^ and industrial scales^24^ to validate the operations and control of MBRs under extreme conditions.

##### 2.4.1.3 Model Selection :

The models selected to represent the knowledge base were based on mathematical calculations for numerical data processing, whereas knowledge‐based techniques were used for the advanced control algorithm and expert supervision simulation.

##### 2.4.1.4 Model Implementation :

The KB was implemented using the 3‐level architecture presented in Comas et al.^25^: (i) data gathering and signal processing, (ii) advanced control system, and (iii) knowledge‐based supervision. The lowest architecture level implements a set of mathematical data verification algorithms to identify data errors (missing data, outliers, etc.) and to perform reconstruction. The middle level includes the control logics of the advanced control system, which are implemented as a decision tree^24^ that involved a series of control actions for energy optimization based on the temporal evolution of MBR performance as specified by the nutrient removal efficiency, biomass characteristics, and permeability trends. Finally, the top level consists of a set of knowledge‐based rules that supervise the control module and regulates the module in case of operational problems, start‐up phases, and mechanical equipment or electrical malfunctions and/or failure.

##### 2.4.1.5 Validation :

The COLMATAR EDSS was successfully validated for long‐term operations in three pilot plants and a full‐scale WWTP (La Bisbal d'Empordà); thus, the EDSS covered a wide range of design and operational conditions (e.g., wastewater type, plant size, hydraulic conditions, biomass characteristics, operational flux, MBR and membrane configuration, and surface). The EDSS was shown to be a reliable and robust tool capable of optimizing the performance of MBR processes and reducing operational costs, as it decreased around 30% and 20% (on average) of the energy required to operate the pilot‐scale and full‐scale plants, respectively,^23^, ^24^ and presented a corresponding reduction of CO_2_ emissions. Although the main objective was to reduce energy consumption, the EDSS also improved the online fouling monitoring, reduced the chemicals consumption and overall increased the reliability and efficiency of the wastewater treatment process.

#### 2.4.2 Use and Transfer Evolution

The research project funding enabled the development of the proof of concept of the tool and the sequential development, implementation and validation of the EDSS at the pilot plant, both on a laboratory and an industrial scale. The successful results achieved at the pilot scale together with the identification of market needs for such technology directed our efforts to the improvement of the academic EDSS prototype so that a viable technical and commercial product could be developed. This step was clearly facilitated by two new projects supporting technology transfer. The goal was twofold: first, the technical objective was to finalize a robust EDSS and valorize it through a proof of concept of the tool's benefits at the full scale over long‐term periods (two years); second, the commercial objective was to foster communication and transfer the plans for the EDSS to the market. Both the research group and the company jointly conducted these valorization projects.^26^

The EDSS was protected by a Spanish patent (ES 2333837B1) owned equally by the university and the company, and the name for the commercial product was registered as SmartAirMBR. The valorization projects also enabled the evaluation of different alternatives for patent marketing and the organization of showcase meetings with water resource managers. Based on our previous experience, the best solution included locating an industrial partner working on process automation and control who was interested in exploiting the patent. Finally, the research group and the company received another technology transfer grant to demonstrate the viability of the EDSS in other countries at different configurations and scales and to further explore collaboration projects.

The market demand together with the feasibility illustrated at the full scale and the absence of close competitors suggests that the COLMATAR EDSS has a significant potential for commercial development because it provides a good solution for an important wastewater treatment problem at a competitive investment cost, and it does not require additional equipment and has an estimated return period of two to three years.

## 3 Discussion

**Figure 1:**
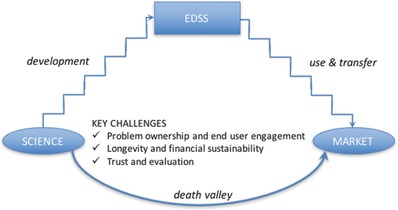
EDSS: from science to market with expected death valley.

As previously mentioned, the use and transfer step is complex and involves interrelated critical challenges. In this section, our experience is discussed according to the three primary challenges identified in the introduction (**Figure** **1**): (i) identifying problem ownership and engaging the end‐user, (ii) encouraging longevity and financial sustainability, and (iii) trusting and evaluating the system.

### 3.1 Problem Ownership and End‐User Engagement

The problem owner depends on the problem the EDSS is trying to solve. Sometimes, it will be a utility for operational and maintenance problems, sometimes a water agency for cost‐benefit problems or sustainability, but it could also be a regulating agency for political transparence. The identification of the problem owner, which might be different of the one affected by the problem, and his/her recognition of ownership is essential to take actions to solve the problem. The mental framework of each owner is different since they have different goals and different procedures to achieve them. We do not think a generic recipe is valid but it will depend on each situation. However, we propose some general criteria.

Utilities have usually very specific information from their installations, which allows specific knowledge to be acquired after high efforts, due to the paradox of expertise.^27^ Besides, there is a difficulty on generalizing this expert knowledge. However, its implementation and use is guaranteed since the knowledge base fits very well with the problem requirements. This is typically the case for EDSS designed for the management of water infrastructures.

For water agencies, there is a similar problem regarding the specificity but different in terms of the implementation and use. In this case, EDSS are usually developed as tools for planning. The identification of the problem owner in the PSARU EDSS was excellent, allowing its direct involvement and achieving great trust in the tool. However, the transfer step to similar problems was not done due to the specificity of the problem and the lack of interest of the problem owner in gaining any additional benefit beyond the resolution of their problems.

A regulator can be an excellent problem owner if it identifies EDSS as standardized tool that can be useful for their work. In this case, the status of independent entity that makes these tools can be very useful because they allow regulators, as independent entities, to negotiate with their interlocutors in a more systematic way, justifying their decision‐making.

A consulting engineer may be the optimal problem owner when the objective is a massive application of an EDSS. That is the case for the ATL EDSS, since it has been implemented in various facilities all over the world. From the developer's point of view, this application might present some limitations because consultant engineer usually are interested in simplified and generic tools, which sometimes contradicts with the more systematic academic will.

End‐user engagement is typically considered a key element for successfully applying EDSSs^28^ because it can elicit relevant information and knowledge and enable easier adoption by reducing the cost and effort related to training and dissemination. However, according to our experience, such outcomes do not always occur. Only one out of the four case studies presented in this paper followed this ideal engagement scheme perfectly, and another case partially followed this scheme; however, the remaining cases exhibited different characteristics.

For the PSARU EDSS (see Section 2.1), user involvement was important. A clear champion within the institution was identified who could manage the challenge of selecting the most adequate WWTP for small communities using an innovative recommendation approach. This solid engagement also enabled the incorporation of relevant information and facilitated the adoption of the EDSS within the commissioning authority.

The COLMATAR EDSS (Section 2.4) was a private end‐user driven approach. In this case, the driving force was shared with the university (developer), who had an interest in developing a practical tool but also in progressing the fundamental knowledge and tools within the MBR scientific field. In addition, the end‐user wanted to solve a new problem with a new mentality by developing a smart system for the improvement of MBR control. This involvement enabled the identification of relevant information, and when the company realized that there was a business opportunity, it also enabled the development of a business plan to optimize the implementation costs for other end‐users.

Nevertheless, end‐user involvement cannot be guaranteed in all cases, particularly when the users do not properly identify or explain the problem or they do not take risks with new approaches to solve new problems, which occurred for both the ATL EDSS (Section 2.2) and NOVEDAR EDSS (Section 2.3). In the former, the application of artificial intelligence techniques was not considered feasible for the improvement of activated sludge process operation and control. Despite its complexity, neither the authorities nor the operating companies felt that the investment of resources and time to develop an EDSS was worthwhile. However, academia perceived that EDSSs were needed to improve process supervision and began their development. Therefore, the end‐user involvement in this case was not absent, although it was reactive to the developers' demand rather than pro‐active, and they only participated in meetings and replied to questionnaires and did not represent a critical factor. Instead, the university determined the road map. A similar situation occurred with the NOVEDAR EDSS, which involved eleven European universities and several companies. However, from the very beginning of the project, it was evident that an academic product would be delivered. For these two case studies, if real engagement did not occur at the beginning of EDSS development, then interest in the information included in the tool could not be guaranteed. Moreover, the adoption of these two EDSSs was also more difficult because they were initially validated primarily by academics, although the case studies evaluated were real.

### 3.2 Longevity and Financial Sustainability

Although the longevity challenge could be related a priori to end‐user engagement (because it can be used to obtain a tool that provides relevant information, is easily adopted and has a lower promotional cost), our experience indicates that reality does not necessarily follow this pattern. The evolution of the four EDSSs allowed for the identification of different situations regarding the relationship between longevity and end‐user engagement.

For the PSARU EDSS, although a champion was identified and strong user engagement occurred during the development phase, the longevity was limited. Once the EDSS resolved the problem that motivated its development, the water agency was not interested in continuing supporting the knowledge base update, commercializing the EDSS or finding a company that could perform the commercialization. Although the promotional phase would not have been particularly costly, it was not among the water agency's objectives, and outside companies considered the EDSS to be too problem specific. Therefore, a business plan was never developed for this application.

The driving force for both the NOVEDAR EDSS and ATL EDSS was identifying the problem to be solved by the research groups, particularly when the problem was not recognized by practitioners (utilities and companies). The advantage of a research‐driven approach is the possibility of evaluating multiple alternatives, which may include risky, time consuming, or even nonviable alternatives by the end‐users. Such situations enable the development of ambitious EDSSs that initially might not find demand among end‐users but can have a longer service life and later dissemination. Therefore, although the adoption process was more expensive and longer, the longevity of the EDSS offered higher guarantees.

The first business plan for the ATL EDSS developed by the researchers was rejected because the prototype was built from an academic perspective. Thus, a second business plan was required, and the end‐users ultimately turned to entrepreneurs to adapt the prototype for practical implementations.

The commercialization step was easier for the NOVEDAR EDSS because private company decided to license it after the prototype was carefully analyzed using different evaluation protocols. Thus, the company itself developed the business plan according to its specifications. An intermediate situation occurred for COLMATAR EDSS because the business plan was developed by the company and the university together.

In our experience, end‐user engagement may certainly help to ensure EDSS longevity; however, involvement should occur in the development and application phases as well as in the commercialization and dissemination phases. If the idea is good enough, then researchers can become the driving force and achieve product longevity, even if the adoption process is longer. This pathway requires a higher *activation energy*, although it might provide a more favorable outcome.

On the other hand, the existence of incentives to end‐users to incorporate innovations such as EDSS or tools for optimization in general is a key point to ensure EDSS longevity and financial sustainability. There should be an adequate regulatory or economic framework favoring those utilities or authorities who take some risks and implement innovations in field. Otherwise, if economic or governance schemes do not promote incentives, because operating companies have too short time to recover the investment, EDSS will hardly be used. For example, water utilities often sign exploitation contracts with a short time frame, which do not facilitate any investment in innovation, such as on implementing mathematical models, EDSS or other support tools for process or design optimization. In any case, EDSS can incentivize better than other tools (sensors, conventional control systems) because they can justify economic or energy savings and, in addition, the reasons of the decisions taken can be rationalized. In this way, the tool fosters the end‐user confidence in the system. In fact, if correctly developed and implemented, EDSS can in theory provide more reliable decisions that potentially subjective decisions that humans could make when dealing with a complex problem.

### 3.3 Trust and Evaluation

Confidence in EDSS and between EDSS developers and end‐users is fundamental, very difficult to gain but easy to lose. Besides, no clear mechanisms/methodologies to obtain it exists since, in some cases, this can be obtained quickly, while in others it has appeared after a longer experience of joint collaboration in previous studies between academia and water agencies, probably not related to EDSS development. That was the case for the PSARU EDSS.

The EDSS capability on providing justifications for the reasoning processes used to carry out diagnosis, to look for the causes of the problem, to find solutions and to, finally, propose different alternatives considerably helps on improving the trust during EDSS operation. This is especially true for those EDSS involving intelligent deliberative components, which use a latent reasoning mechanisms, such as the rule‐based reasoning systems or case‐based reasoning systems, which enable to generate explanations about the reasoning processes that promote the end‐user trust on the EDSS results.^29^, ^30^

Finally, the EDSS validation is a very important step to enhance end‐users confidence. When end‐users are involved from the very beginning, as it was the case for the ATL EDSS, a continuous evaluation of the different ongoing EDSS prototypes was carried out, so that trust on the decision proposed by the final version of the EDSS is guaranteed. On the contrary, when end‐users are not involved from the very beginning, either end‐users are fully involved in the final validation step or validation results are perceptibly shown to them. For the NOVEDAR EDSS, for example, a well‐defined procedure involving several meetings with the different departments of the company were needed before trust on the tool results was assured.

Even the most advanced EDSS can also integrate traditional design or operational guidelines as part of the knowledge base to reproduce conventional or current practices carried out by human experts. That would also foster confidence of end‐users to EDSS results. However, as today, EDSS can only provide support to decision‐making. The final responsibility never belongs to the EDSS tool but to the person(s)/actors using it and making decisions based on its outputs, i.e., water authorities or companies operating wastewater infrastructures. Normally, this should be specified in the agreement between EDSS developers and final users.

Regarding the evaluation challenge, the four EDSSs were successful with respect to user satisfaction, although this success occurred at different steps in the development process. For PSARU EDSS, the client was satisfied with the results because the EDSS was finally applied to select the most adequate wastewater treatment for approximately 3500 small communities. It is difficult to determine whether the results provided by the EDSS would have been the same as those provided by a set of experts facing the same problem; however, the procedure was able to provide standardized proposals that were greatly appreciated by the end‐user (a public administration).

For the ATL EDSS, the initial prototype presentations to the potential clients were not satisfactory because the clients indicated that the analysis of the activated sludge operational problems conducted by the EDSS were too complex and time consuming. When the system was adapted to actual situations by practitioners instead of researchers, all of the different evaluation criteria were positive. Currently, more than one hundred WWTP managers worldwide rely on the ATL EDSS to improve activated sludge process supervision and meet effluent water quality regulations with minimum energy consumption.

NOVEDAR EDSS and COLMATAR EDSS have successfully passed client examinations for implementation and generalization; therefore, we are confident that their successful application and adoption will occur in the near future.

### 3.4 Sociocultural Contexts

A close relationship and trust between EDSS developers and local end‐users will guarantee the avoidance of sociocultural problems. However, this relationship sometimes is not so good or simply does not exist. For example, when aiming at developing EDSS to solve generic environmental problems taking place in different parts of the world. In the latter case, the aim should be to develop EDSSs generic enough which do not conflict with sociocultural aspects of the application country. Indeed, an important part of the phenomena related to wastewater management can be generalized and, as such, must be described in EDSS by means of WWTP ontologies.^31^ For example, the fact that MBR produce higher effluent quality than CAS can be understood similarly in Catalonia as in Japan. However, there are always some local aspects or specificities to take into account when taking decisions that EDSS should also take into account. These local criteria must be defined within the EDSS as customizable parameters based on the local technical, economic, environmental, or political criteria. For example, different environmental regulations might apply for different countries. The NOVEDAR EDSS includes by default the Spanish legislation (transposed from European Directives) but this type of information is parametrizable so that it can be customized or modified when applied to another country. The ATL EDSS for online control system of conventional WWTPs is a good example since, even though developed in Catalonia from the academia, with the involvement of local end‐users, it is nowadays implemented in Poland, England and Chile, among nine more other countries around the globe. Sociocultural aspects can also be easily considered in EDSS for planning purposes by modifying the assigned weights of the existing social criteria (increasing or decreasing depending on the local relevance) or, if needed, by adding more specific criteria. See the case for NOVEDAR EDSS, which has also been validated with case studies from US.^32^ Therefore, transferability of our EDSS is easy and has already been demonstrated, since a significant part of the knowledge base can be generalized while the specific part related to sociocultural difference not yet but we foresee that being easy as well.

## 4 Conclusions

Although each analyzed case was different, a set of common traits could be identified and generalized in these EDSS use and transfer processes. In all cases, we identified situations considered to be the death valley for the product, which is the moment when a product developed by academics has reached a level of maturity such that researchers are not interested in its further development, but it is still not marketable. New stages should be defined to achieve this goal.

For the PSARU EDSS, because the administration did not provide a driving force, the product did not succeed in crossing the death valley. For the ATL EDSS, the university entrepreneur did not have enough influence, and the product was at risk of not crossing the death valley. However, this stage was successfully overcome because of the commercial interest of new partners, which permitted the establishment of a significant synergy (pulling it from the market). For the NOVEDAR EDSS, the death valley will be crossed with a private company that is assuming the cost of implementing the market requirements for the EDSS and commercializing the process. This situation appears to be similar to what occurs with the mathematical models of WWTPs or environmental systems in general, which has been recently noted by Robson.^33^

The role of the end‐user and the identification of an internal champion are positive for certain aspects pertaining to development and adoption; however, they also may introduce limitations to the process and do not guarantee the longevity of the tool.

Our experience demonstrates that the driving force can come from academia, which will increase the time and effort required to reach a final product. However, a research‐driven approach enables the implementation of wider perspectives when evaluating different alternatives, which may be helpful for ensuring the successful application and final outcome of the EDSS and could be constrained for projects directed by end‐users. Although it seems contradictory, in certain cases, a lack of end‐user involvement at the beginning of the process can be beneficial over the long term.

The existence of incentives to end‐users to incorporate innovations such as EDSS or tools for optimization in general is a key point to ensure EDSS longevity and financial sustainability. Besides, a successful validation and end‐users confidence on the EDSS results is indispensable for successful market application and adoption. Another positive element is the accelerated improvement in available instrumentation (more data and more reliable), faster computation and better and faster connectivity that will allow the implementation of better EDSS in many communities at a cheaper price.

In summary, we suggest that the construction of a successful EDSS should focus significant efforts on crossing the death valley toward a general use implementation by society (the market) rather than on development (climbing down). The final step has become the major task (hill climbing) to obtain a useful EDSS that can be used by the public. Thus, the death valley has shifted from the development step to the use and transfer step.
